# Influence of Coal Petrology Characteristics on the Organic Matter Adsorption Properties: A Molecular Simulation Perspective

**DOI:** 10.3390/ijms27031385

**Published:** 2026-01-30

**Authors:** Qingfeng Lu, Wenfeng Wang, Penghui Bo, Bo Zhu, Fengjun Shao

**Affiliations:** 1 Jiangsu Key Laboratory of Coal-Based Greenhouse Gas Control and Utilization, Carbon Neutrality Institute, China University of Mining & Technology, Xuzhou 221008, China; 2 Key Laboratory of Coalbed Methane Resources & Reservoir Formation Process, Ministry of Education, School of Resources and Geosciences, China University of Mining & Technology, Xuzhou 221116, China; bopenghui123@163.com (P.B.); zhubo04321@163.com (B.Z.); 3 Institute of Geology and Mining Engineering, Xinjiang University, Urumqi 830002, China; shaofj0805@xju.edu.cn

**Keywords:** coal macerals, molecular dynamics simulation, adsorption–desorption dynamics, residence time, hydrogen bonding

## Abstract

The interaction between small organic molecules and coal macerals plays a critical role in regulating fluid retention and transport in coal-related energy and environmental systems. However, the microscopic mechanisms governing adsorption selectivity and interfacial dynamics on different maceral surfaces remain insufficiently understood. In this study, molecular dynamics simulations were employed to investigate the adsorption and desorption behaviors of toluene (TOL) and tetrahydrofuran-2-ol (FUR) on inertinite (INE) and vitrinite (VIT) surfaces at the molecular level. Time-dependent variations in adsorption number, residence time, molecular mobility, interaction energies, and hydrogen-bond characteristics were systematically analyzed. The results reveal strong maceral- and molecule-dependent adsorption preferences. TOL exhibits the most stable adsorption on the INE surface, characterized by rapid surface accumulation, minimal desorption, and a long residence time of 0.43547 ns, which is mainly driven by strong van der Waals interactions and aromatic stacking effects. In contrast, TOL adsorption on VIT is highly dynamic, with frequent desorption events and a markedly reduced residence time of 0.1077 ns. FUR shows relatively weaker and more reversible adsorption on INE, accompanied by enhanced molecular mobility and a shorter residence time of 0.31354 ns. Notably, FUR demonstrates stronger surface retention on VIT, with an extended residence time of 0.34439 ns, which can be attributed to increased electrostatic contributions and intermittent hydrogen bonding. Hydrogen-bond analysis indicates that FUR forms longer-lived hydrogen bonds with VIT (22.05 ps) than with INE (17.86 ps), providing additional stabilization at the interface. These findings elucidate the distinct adsorption mechanisms of aromatic and polar molecules on heterogeneous coal macerals and offer molecular-scale insights into organic matter–coal interfacial processes relevant to energy extraction and subsurface transport.

## 1. Introduction

Coal-bearing formations contain abundant organic matter and complex pore–surface structures, making them important reservoirs for the storage and transport of organic compounds. Interactions between coal surfaces and organic molecules play a crucial role in a variety of geological and engineering processes, including coalbed methane accumulation, organic contaminant retention, and hydrocarbon migration in unconventional reservoirs [1,2,3,4]. In such systems, adsorption at coal interfaces largely controls the distribution, mobility, and availability of organic molecules, thereby directly influencing resource recovery efficiency and environmental risk. Unlike ideal mineral surfaces, coal exhibits pronounced chemical and structural heterogeneity, which gives rise to diverse adsorption behaviors that cannot be fully captured by macroscopic thermodynamic descriptions. Consequently, elucidating the molecular mechanisms governing organic molecule adsorption on heterogeneous coal surfaces remains a fundamental challenge [5,6,7,8].

Coal macerals exert a strong influence on the physicochemical properties of coal, particularly its adsorption behavior. Among them, vitrinite and inertinite are two dominant macerals that differ markedly in molecular structure and surface chemistry [9]. Previous structural characterizations using techniques such as elemental analysis, XPS, FTIR, and solid-state ^13^C NMR have shown that vitrinite is enriched in aliphatic carbon and oxygen-containing functional groups, whereas inertinite exhibits a higher degree of aromatic condensation and coalification [10]. These compositional differences are expected to affect molecular-scale interactions with external adsorbates. Previous studies have shown that maceral composition is often accompanied by differences in pore structure and surface exposure, which may influence adsorption behavior at larger scales [11,12,13,14,15]. In addition, theoretical studies based on density functional theory have demonstrated that maceral surface chemistry can strongly regulate adsorption selectivity, such as the preferential adsorption of CO_2_ over CH_4_ on vitrinite models [16]. While gas adsorption studies have provided important insights into coal–fluid interactions, the adsorption behavior of liquid and low-molecular-weight organic compounds involves additional complexities. Compared to simple gas molecules, organic liquids are characterized by larger molecular size, structural flexibility, and diverse functional groups, which enable multiple interaction mechanisms, such as π–π stacking, hydrophobic interactions, and hydrogen bonding. As a result, adsorption trends inferred from gas systems cannot be directly extrapolated to organic molecules. Most existing studies focus on bulk coal samples or isolated structural features, while comparative investigations of adsorption behavior on individual maceral surfaces at the molecular scale remain limited.

Coal reservoirs also interact with a wide range of organic small molecules present in formation fluids, hydrocarbon mixtures, and coal-derived byproducts [17,18,19,20,21]. Experimental and simulation studies have shown that aromatic and polar organic molecules can adsorb onto coal surfaces through mechanisms such as π–π interactions, hydrophobic effects, and hydrogen bonding, and that these interactions can significantly modify interfacial properties and adsorption dynamics [22,23,24,25]. However, previous work has largely emphasized gas adsorption or simple hydrocarbons, whereas the adsorption behavior of polar organic molecules on different coal macerals has received comparatively little attention. In particular, how maceral heterogeneity and molecular polarity jointly influence adsorption selectivity and interfacial dynamics remains insufficiently understood [26,27,28,29,30,31].

Despite extensive studies on coal–fluid interactions, several critical gaps remain at the molecular scale. Existing investigations predominantly focus on gas adsorption or bulk coal samples, while maceral-specific adsorption behaviors of liquid organic molecules are rarely quantified under identical conditions. In particular, the combined effects of maceral type and organic molecular polarity on adsorption stability, interfacial dynamics, and residence behavior have not been systematically examined. To address these gaps, this study employs molecular dynamics simulations to systematically investigate the adsorption behavior of two representative organic molecules—toluene and tetrahydrofuran-2-ol—on vitrinite and inertinite surfaces. Toluene serves as a typical nonpolar aromatic molecule, while tetrahydrofuran-2-ol contains both a heterocyclic structure and a polar hydroxyl group, enabling a direct comparison of polarity-dependent adsorption mechanisms. By analyzing adsorption configurations, interaction energies, residence times, and interfacial dynamics, this work aims to clarify how maceral type and organic molecular properties jointly govern adsorption processes at the molecular level, thereby providing new insights into organic matter–coal interfacial behavior relevant to subsurface energy and environmental systems. It should be noted that this work focuses on molecular-scale adsorption mechanisms on idealized maceral surfaces, with the aim of isolating the effects of surface chemistry and molecular interactions, rather than explicitly resolving pore network effects.

## 2. Results and Discussion

**Figure 1:**
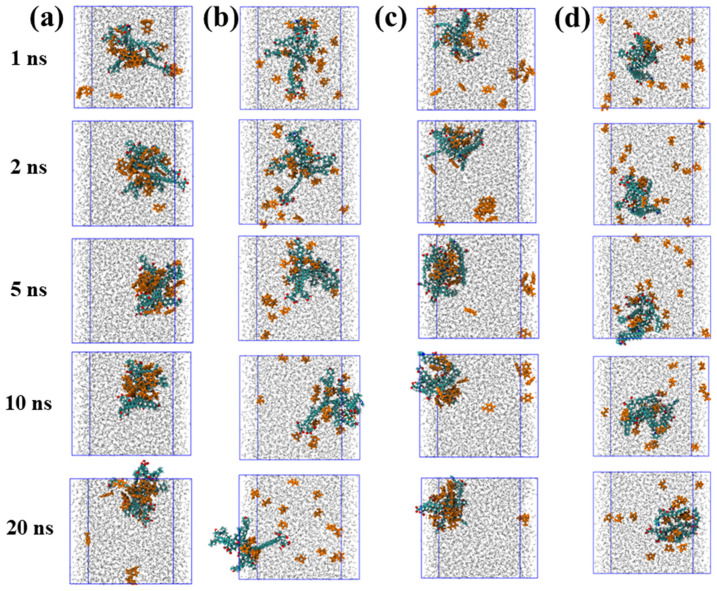
Adsorption structure evolution of (**a**) Toluene/(**b**) Tetrahydrofuran-2-ol on inertinite surface and of (**c**) Toluene/(**d**) Tetrahydrofuran-2-ol on vitrinite surface.

To elucidate the dynamic adsorption behavior of organic molecules on coal maceral surfaces, the time evolution of TOL and FUR adsorption on INE and VIT was systematically examined. The adsorption behavior of TOL on the INE surface exhibits a clear time-dependent evolution (Figure 1a). At the early stage of the simulation (≈1 ns), TOL molecules dispersed in the aqueous phase gradually migrate toward the INE framework, driven primarily by π–π interactions between the aromatic rings of TOL and the condensed aromatic backbone of INE. At this stage, a small number of TOL molecules remain solvated in the bulk solution. By 2 ns, the majority of TOL molecules are adsorbed onto the INE surface, preferentially associating with the aromatic skeletal regions rather than surface functional groups. As the simulation progresses to 5 ns, the adsorbed TOL molecules exhibit closer packing and enhanced surface affinity, accompanied by a tendency to form surface-associated aggregates. However, this compact adsorption configuration gradually relaxes at later stages. Around 10 ns, the initially dense adsorption layer becomes less ordered, and partial desorption is observed by 20 ns, indicating a dynamic equilibrium between surface-bound and solvated TOL molecules. In contrast, FUR displays a markedly different adsorption pattern on the INE surface (Figure 1b). Although FUR molecules also migrate toward the INE framework during the initial stage (≈1 ns), their accumulation rate is noticeably slower than that of TOL. By 2 ns, only a fraction of the molecules are adsorbed onto the surface. Throughout the remainder of the simulation up to 20 ns, FUR maintains a relatively loose and dispersed adsorption state, without forming the compact adsorption layer observed for TOL. This behavior suggests weaker surface affinity and limited cooperative interactions, likely arising from the reduced aromatic character and the competing influence of polar functional groups interacting with the surrounding aqueous environment.

The adsorption behavior of TOL on the VIT surface shows a progressive and irreversible character (Figure 1c). Owing to the predominantly aromatic nature of the coal molecular framework, TOL molecules readily interact with the VIT surface through aromatic–aromatic interactions. At approximately 1 ns, a portion of TOL molecules has already migrated from the aqueous phase and accumulated on the VIT surface. By 2 ns, in addition to the surface-adsorbed TOL, the remaining TOL molecules dispersed in water begin to associate with each other, forming transient molecular clusters. As the simulation proceeds, these clusters gradually disintegrate and migrate toward the VIT surface, ultimately resulting in complete adsorption of TOL without observable desorption events throughout the simulation period. This behavior suggests a strong and persistent affinity of TOL for the VIT surface. In contrast, the adsorption of FUR on the VIT surface follows a distinctly different pathway (Figure 1d). Rather than being driven by aromatic interactions, FUR adsorption is primarily governed by specific interactions between its polar functional groups and the surface functionalities of VIT. Consequently, the adsorption process occurs at a noticeably slower rate. Unlike TOL, FUR does not exhibit clustering behavior either on the VIT surface or in the aqueous phase. Even at 20 ns, a considerable fraction of FUR molecules remain dispersed in the bulk water, indicating weaker surface affinity and a more dynamic adsorption–desorption balance compared to TOL.

**Figure 2:**
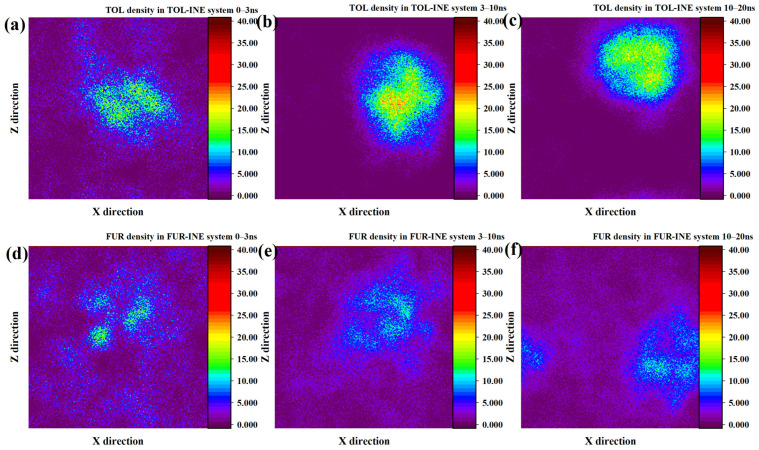
Two-dimensional density distribution contour plots of TOL adsorbed on the INE surface at (**a**) 0–3 ns, (**b**) 3–10 ns, and (**c**) 10–20 ns and FUR adsorbed on the INE surface at (**d**) 0–3 ns, (**e**) 3–10 ns, and (**f**) 10–20 ns.

To further resolve the spatial evolution of adsorption on the INE surface, two-dimensional number density maps were analyzed to track the time-dependent distribution of TOL and FUR molecules. The two-dimensional density evolution of TOL on the INE surface reveals a rapid and pronounced aggregation process. Within the initial 0–3 ns, TOL molecules exhibit a clear tendency to accumulate near the INE surface, indicating a strong driving force for surface association. During the subsequent 3–10 ns period, the majority of TOL molecules become adsorbed onto the INE surface and progressively assemble into compact clusters. This aggregation is reflected in the density maps by a pronounced central high-density region, with density gradually decreasing toward the periphery, giving rise to a quasi-spherical distribution pattern. Such features suggest cooperative π–π interactions among TOL molecules combined with strong affinity toward the aromatic backbone of INE. As the simulation advances to 10–20 ns, a slight reduction in central density is observed, consistent with partial desorption inferred from the structural evolution discussed above. Nevertheless, most TOL molecules remain confined near the INE surface, indicating that desorption is limited and does not disrupt the overall adsorption layer (Figure 2a–c). In contrast, the density evolution of FUR on the INE surface is characterized by a much slower and more dispersed aggregation behavior. During the initial 0–3 ns, only a small fraction of FUR molecules adsorb onto the INE surface, forming localized and weakly defined density maxima. From 3 to 10 ns, these high-density regions expand only marginally and then remain largely unchanged until the end of the simulation. Unlike TOL, FUR does not develop a pronounced spherical or clustered density pattern, reflecting the absence of strong intermolecular association and weaker cooperative interactions on the INE surface. This behavior can be attributed to the dominant role of polar functional groups in FUR, which preferentially interact with the surrounding aqueous environment rather than promoting surface-driven aggregation (Figure 2d–f) [32,33,34,35,36,37,38].

**Figure 3:**
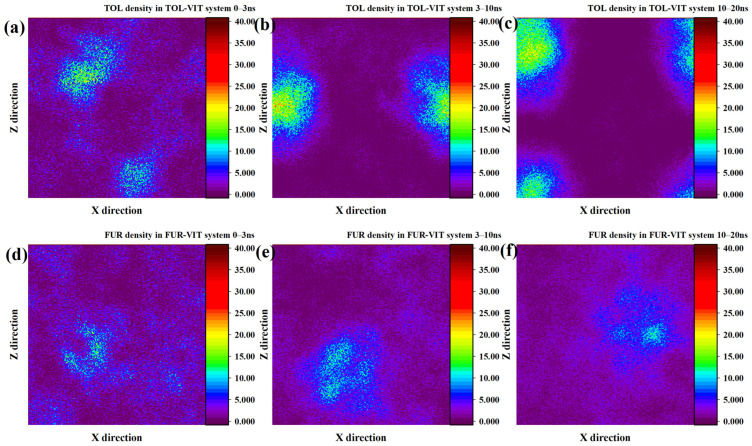
Two-dimensional density distribution contour plots of TOL adsorbed on the VIT surface at (**a**) 0–3 ns, (**b**) 3–10 ns, and (**c**) 10–20 ns and FUR adsorbed on the VIT surface at (**d**) 0–3 ns, (**e**) 3–10 ns, and (**f**) 10–20 ns.

Compared with its behavior on the INE surface, the adsorption of TOL on VIT proceeds at a relatively slower pace and exhibits a distinct density evolution pattern. During the initial 0–3 ns, the adsorbed TOL molecules are distributed in a relatively dispersed manner, giving rise to two loosely defined high-density regions on the VIT surface rather than a single compact cluster. At this stage, no well-developed spherical density feature is observed, indicating limited cooperative aggregation. As the simulation progresses into the 3–10 ns interval, these two diffuse density regions gradually merge and reorganize into a more compact, quasi-spherical aggregation structure. From this point until the end of the simulation, the high-density region continues to expand and intensify, suggesting enhanced molecular packing and stronger surface confinement, which reflects the progressive stabilization of TOL adsorption on the VIT surface (Figure 3a–c). In contrast, the density evolution of FUR on the VIT surface indicates considerably weaker adsorption. Within the first 0–3 ns, FUR molecules show little tendency to accumulate near the surface, and no distinct density maxima are formed. Only during the 3–10 ns period does a limited degree of local aggregation emerge, characterized by small and weakly defined density regions. However, this aggregation remains unstable, and by 10–20 ns the density distribution becomes increasingly diffuse, particularly on one side of the surface, reflecting a loosely bound adsorption state. Such behavior highlights the limited affinity of FUR for the VIT surface and suggests that polar functional group interactions are insufficient to induce sustained surface aggregation in the absence of strong cooperative effects (Figure 3d–f).

**Figure 4:**
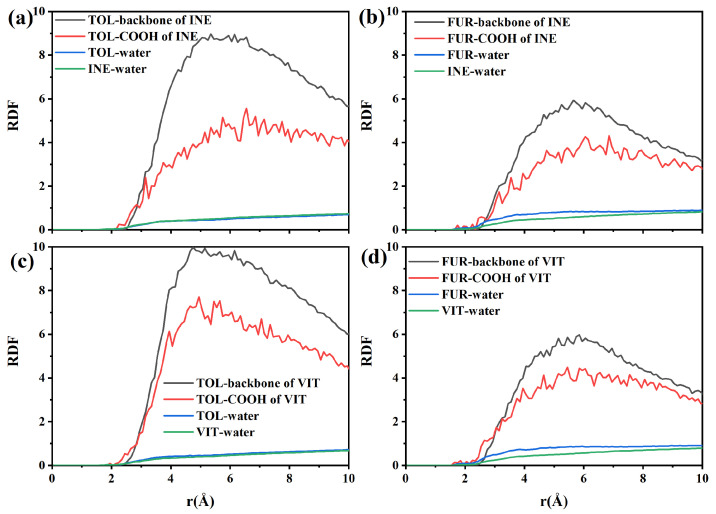
Radial distribution functions between components in the (**a**) TOL/(**b**) FUR-INE system and in the (**c**) TOL/(**d**) FUR-VIT system.

To quantify the intermolecular interactions governing adsorption, radial distribution functions (RDFs) were calculated for TOL and FUR in the vicinity of INE and VIT surfaces. As shown in the RDF between TOL and the INE backbone, a pronounced first peak emerges at approximately 2.6–2.8 Å, followed by a rapid increase in g(r) that remains at a relatively high level (g(r) > 4) over the 3.5–5.0 Å range (Figure 4a). This short-range enrichment indicates a strong tendency of TOL molecules to reside in close proximity to the carbonaceous backbone of INE, consistent with direct surface adsorption rather than weak, solvent-separated association. The absence of a comparable peak at larger distances further suggests that the interaction is dominated by short-range forces, primarily driven by π–π stacking and hydrophobic interactions between the aromatic rings of TOL and the aromatic-rich INE framework. In contrast, the RDF between TOL and the carboxyl groups of INE exhibits a noticeably weaker first-shell structure, with a much lower peak intensity and a delayed rise in g(r). This indicates that polar functional groups play a secondary role in stabilizing TOL at the INE surface, and that TOL preferentially avoids strong localization near oxygen-containing sites. Meanwhile, the RDF of TOL–water shows a relatively smooth and low-amplitude profile, lacking a sharp first peak, implying that hydration interactions are largely disrupted upon adsorption. Combined with the steadily increasing RDF between INE and water at larger distances, these results suggest that TOL adsorption effectively displaces interfacial water molecules and is therefore thermodynamically favored through hydrophobic dehydration and aromatic affinity rather than specific polar interactions.

Compared with TOL, the radial distribution functions reveal a distinctly different interaction pattern between FUR and the INE surface (Figure 4b). The RDF between FUR and the INE backbone shows a delayed and less intense first coordination shell, with the first noticeable rise appearing at approximately 2.8–3.0 Å, followed by a broad and relatively smooth maximum in the 3.0–4.5 Å range. The peak intensity remains moderate (g(r) ≈ 2–3) and does not exhibit a sharp, well-defined first maximum, indicating that FUR does not strongly localize on the aromatic backbone of INE. This weak short-range ordering suggests that π–π interactions play only a limited role in stabilizing FUR near the INE surface. In contrast, the RDF between FUR and the carboxyl groups of INE displays a more pronounced and persistent enhancement at short distances. A clear first-shell feature emerges around 2.4–2.6 Å, followed by a gradual increase in g(r) that remains consistently higher than that of the backbone interaction over the entire distance range. This behavior indicates a preferential association of FUR with polar functional groups, which can be attributed to hydrogen bonding and dipole–dipole interactions involving the hydroxyl group of FUR. However, the absence of a sharp, high-amplitude peak suggests that these interactions are dynamic and transient rather than forming stable, well-defined adsorption sites. The RDF of FUR–water further supports this interpretation, as it exhibits a continuously increasing profile without a clear depletion region near the surface. This implies that FUR maintains substantial hydration even when approaching the INE surface, in contrast to the dehydration-driven adsorption observed for TOL. Meanwhile, the steadily increasing RDF between INE and water indicates that water molecules remain abundant at the interface, reinforcing the notion that FUR adsorption does not significantly displace interfacial water. Overall, these RDF features demonstrate that FUR–INE interactions are governed by weak, competition-driven polar interactions, leading to loose and reversible adsorption rather than compact surface binding.

The radial distribution functions further elucidate the adsorption characteristics of TOL on the VIT surface (Figure 4c). The RDF between TOL and the VIT backbone exhibits a pronounced and well-defined first coordination shell, with the initial sharp increase occurring at approximately 2.6–2.8 Å and a clear maximum located around 3.3–3.6 Å. The peak intensity of this first shell is significantly higher than unity and continues to increase with distance, indicating strong short-range ordering and a high probability of TOL molecules residing near the aromatic backbone of VIT. This behavior reflects the dominant role of π–π stacking interactions between the aromatic rings of TOL and the highly aromatic framework of vitrinite, leading to stable and persistent surface association. In comparison, the RDF between TOL and the carboxyl groups of VIT shows only a modest enhancement at short distances, with relatively low peak intensities across the entire distance range. The absence of a sharp first-shell peak suggests that polar functional groups contribute little to the stabilization of TOL adsorption. This observation is consistent with the nonpolar nature of TOL, for which electrostatic interactions and hydrogen bonding are unfavorable, leaving dispersive and aromatic interactions as the primary driving forces. The TOL–water RDF displays a suppressed probability at short distances, indicating partial dehydration of TOL as it approaches the VIT surface. Meanwhile, the RDF between VIT and water increases smoothly with distance, suggesting that although water remains present near the surface, it does not prevent TOL from accessing aromatic sites. Together, these features imply that TOL adsorption on VIT is governed by strong aromatic affinity and dehydration-assisted surface association, resulting in stable adsorption without noticeable desorption, in agreement with the time-dependent structural evolution observed earlier.

The RDF profiles provide further insight into the adsorption behavior of FUR on the VIT surface (Figure 4d). In contrast to TOL, the RDF between FUR and the VIT backbone shows only a moderate enhancement at short distances. The first noticeable increase appears at approximately 2.6–2.8 Å, followed by a broad and relatively low-intensity maximum centered around 3.3–3.6 Å, indicating weak short-range ordering. The absence of a sharp and dominant first coordination peak suggests that direct aromatic stacking between FUR and the vitrinite backbone is unfavorable, reflecting the limited π–π affinity of FUR compared with aromatic hydrocarbons. Meanwhile, the RDF between FUR and the carboxyl groups of VIT exhibits a gradual rise with distance but lacks a distinct first-shell maximum. Although polar functional groups on VIT provide potential interaction sites, the broad and diffuse nature of the RDF indicates that these interactions are transient rather than strongly localized. This behavior implies that hydrogen bonding or dipole–dipole interactions between FUR and VIT functional groups occur intermittently and do not result in persistent surface anchoring. Notably, the FUR–water RDF displays consistently elevated values over the short- and medium-range distances, indicating a strong hydration shell surrounding FUR molecules. In parallel, the VIT–water RDF increases smoothly, confirming the competitive presence of water near the VIT surface. Together, these features suggest that hydration effects dominate over surface affinity, preventing FUR from displacing interfacial water and forming stable adsorption configurations. As a result, FUR remains largely dispersed in the aqueous phase or loosely associated with the VIT surface, in agreement with the weak aggregation and poor surface coverage observed in the structural and density evolution analyses.

**Figure 5:**
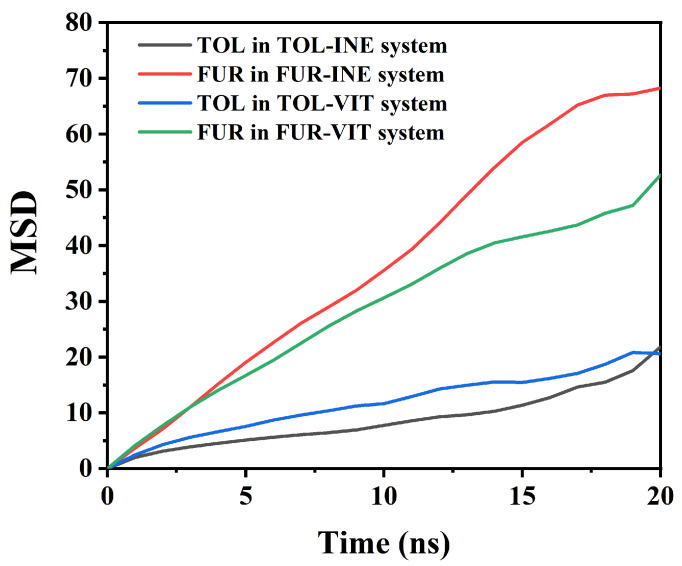
Root Mean Square Displacement of TOL/FUR in Various Composite Systems.

The mean square displacement (MSD) analysis is employed to quantify the dynamic mobility of TOL and FUR in different interfacial systems (Figure 5). By comparing the time-dependent evolution of MSD, the influence of surface affinity and intermolecular interactions on molecular diffusion behavior can be systematically assessed. Based on the MSD data, clear differences in the mobility of TOL and FUR can be identified between the INE and VIT systems, reflecting distinct interfacial interaction strengths and adsorption constraints. In the INE-containing systems, TOL exhibits a relatively slow and nearly linear increase in MSD over time, rising from 0 to 21.84 by 20 ns. The modest slope indicates that the translational motion of TOL is strongly restricted, consistent with stable adsorption of aromatic molecules on the INE surface dominated by π–π stacking and dispersive interactions. In contrast, FUR in the FUR–INE system shows a much steeper MSD growth, reaching 68.23 at 20 ns. This nearly threefold increase compared to TOL suggests significantly higher mobility, implying weaker surface confinement. The polar nature of FUR and its preference for transient, non-specific interactions limit its residence time on the INE surface, resulting in enhanced diffusion. A similar but less pronounced contrast is observed in the VIT systems. TOL in the TOL–VIT system displays intermediate mobility, with the MSD increasing to 20.59 at 20 ns. Compared with TOL–INE, the slightly higher MSD at earlier times indicates weaker adsorption on VIT, consistent with the more heterogeneous surface chemistry and reduced effective π–π overlap. Nevertheless, the overall low MSD values confirm that TOL remains largely surface-associated throughout the simulation. For FUR–VIT, the MSD increases steadily to 52.69 by 20 ns, which is lower than that in the FUR–INE system but still substantially higher than that of TOL. This behavior suggests that although functional groups on VIT can provide localized interaction sites for FUR, these interactions are insufficient to strongly immobilize the molecule, allowing sustained diffusive motion. Overall, the MSD analysis demonstrates that molecular mobility follows the order: FUR–INE > FUR–VIT ≫ TOL–VIT ≈ TOL–INE.

This trend highlights the dominant role of aromatic affinity in constraining TOL motion, while the higher polarity and weaker surface binding of FUR result in enhanced diffusion across both coal maceral surfaces.

**Figure 6:**
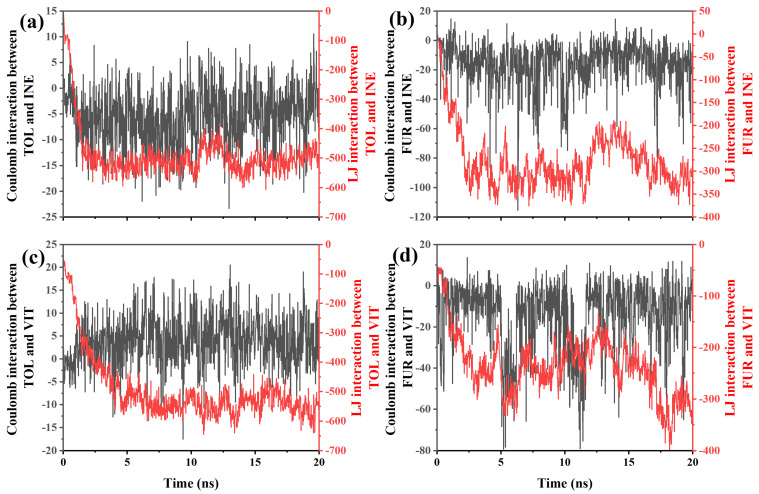
Coulomb and LJ interactions between (**a**) TOL/(**b**) FUR and INE and between (**c**) TOL/(**d**) FUR and VIT.

To further clarify the origin of the distinct adsorption and mobility behaviors, the Coulombic and Lennard–Jones (LJ) interaction energies between TOL/FUR and the INE and VIT surfaces were systematically examined. By decomposing the total nonbonded interactions into electrostatic and van der Waals contributions, the dominant driving forces governing molecular affinity at different coal maceral interfaces can be quantitatively identified. This analysis provides a direct energetic basis for interpreting the observed differences in adsorption stability and dynamic response. Based on the interaction-energy data between TOL and INE (Figure 6a), several clear features can be identified that are consistent with the previously observed adsorption behavior. At the early stage (≈0–1 ns), the Lennard–Jones (LJ) interaction rapidly decreases from about −85 to below −300, indicating that TOL molecules quickly approach and establish close contact with the INE surface. In contrast, the Coulombic interaction remains very weak throughout this period, fluctuating within roughly −10 to 0, suggesting that electrostatic contributions play a negligible role in the initial adsorption. This energy evolution confirms that van der Waals forces, particularly π–π stacking and dispersion interactions between aromatic rings, dominate the early capture of TOL by the INE framework. During the intermediate stage (≈1–5 ns), the LJ interaction further strengthens and stabilizes, reaching values around −500 to −580, while the Coulombic term continues to oscillate around zero with both small attractive and repulsive excursions. The sustained strong LJ attraction reflects the formation of a stable adsorbed configuration, consistent with the aggregation of TOL molecules on the INE surface observed in the structural snapshots and density maps. The minor fluctuations in Coulomb energy arise from transient reorientations of TOL relative to heteroatoms or local charge distributions on INE, but they do not significantly affect the overall binding strength. At longer times (>5 ns), both interaction components exhibit noticeable fluctuations. The LJ interaction remains strongly negative but shows intermittent weakening (e.g., increasing from −580 to −450), while the Coulombic term alternates between weak attraction and slight repulsion. This behavior indicates dynamic rearrangement at the interface and partial detachment–reattachment events of TOL molecules, in agreement with the onset of partial desorption inferred from trajectory analysis. Nevertheless, the persistently large negative LJ energy demonstrates that dispersion interactions continue to dominate the TOL–INE affinity, preventing complete desorption and maintaining an overall adsorbed state.

For the FUR–INE system (Figure 6b), the evolution of interaction energies reveals a markedly different adsorption mechanism compared with TOL–INE, reflecting the polar nature of FUR and its weaker affinity for the aromatic INE surface. At the initial stage (≈0–1 ns), both Coulombic and LJ interactions fluctuate strongly. The LJ term decreases from about −30 to nearly −190, indicating that FUR molecules approach the INE surface and establish short-range contacts. Unlike TOL, however, the Coulomb interaction already shows sizable negative values (down to about −20), together with occasional positive excursions, suggesting frequent reorientation of the polar hydroxyl and ether groups of FUR relative to the heterogeneous charge distribution on INE. This behavior implies that electrostatic interactions start to contribute at an early stage, although they remain highly unstable. During the intermediate period (≈1–5 ns), the LJ interaction further strengthens and reaches values around −300 to −360, but its magnitude is still significantly weaker than that observed for TOL–INE. Meanwhile, the Coulombic interaction becomes more pronounced, frequently ranging from −20 to below −40, with intermittent repulsive events. This combination indicates that FUR adsorption is governed by a mixed mechanism: dispersion forces anchor the molecule near the surface, while transient electrostatic attractions—likely involving hydrogen bonding or dipole–dipole interactions between FUR functional groups and polar sites on INE—continuously reorganize the interfacial configuration. The strong fluctuations are consistent with the loose and dynamic adsorption pattern observed in structural analyses. At longer times (>5 ns), both interaction components remain highly variable. The LJ energy oscillates mainly between −250 and −350, without developing a persistently stronger binding state, while the Coulombic interaction alternates between moderate attraction and occasional strong negative spikes (down to about −50). This sustained variability indicates the absence of a compact, tightly bound adsorption layer. Instead, FUR molecules undergo repeated attachment–detachment and reorientation events, maintaining a relatively mobile interfacial state throughout the simulation.

For the TOL–VIT system (Figure 6c), the interaction-energy evolution indicates a typical nonpolar adsorption process dominated by van der Waals forces, with electrostatic contributions playing only a minor and highly fluctuating role. At the early stage (≈0–1 ns), the Lennard–Jones (LJ) interaction rapidly decreases in magnitude from about −100 to nearly −180, indicating that TOL molecules quickly approach the VIT surface and establish initial contact. In contrast, the Coulombic interaction remains very weak, oscillating close to zero (within approximately −2 to +2). This behavior is expected for TOL, which is nonpolar, and suggests that adsorption is not driven by electrostatics but rather by dispersion interactions associated with π–π stacking and hydrophobic contacts. During the intermediate period (≈1–5 ns), the LJ interaction strengthens significantly, reaching values of about −400 to −550. This continuous decrease reflects progressive packing and reorganization of TOL molecules on the VIT surface, leading to a more compact adsorption configuration. Meanwhile, the Coulomb interaction shows larger fluctuations, occasionally reaching positive values above +5 or negative values below −5, but without a clear trend toward stabilization. These fluctuations mainly arise from transient orientations and local charge inhomogeneities, rather than from any persistent electrostatic binding mechanism. At longer simulation times (>5 ns), the LJ interaction remains strongly negative and relatively stable, mostly ranging between −500 and −600, with occasional deeper minima below −630. This indicates that the TOL–VIT system has reached a stable adsorption state characterized by strong van der Waals cohesion. In contrast, the Coulombic interaction continues to oscillate around zero, with sporadic positive and negative spikes (up to about +17 and down to −10), but its magnitude remains small compared to the LJ term. Therefore, its contribution to the total interaction energy is negligible.

For the FUR–VIT system (Figure 6d), the interaction energies reveal a markedly different adsorption pattern compared with TOL–VIT, characterized by a non-negligible electrostatic contribution superimposed on van der Waals interactions. At the initial stage (<1 ns), both Coulomb and LJ interactions increase rapidly in magnitude. The LJ term decreases from about −54 to nearly −170, indicating the approach and initial anchoring of FUR on the VIT surface. Meanwhile, the Coulomb interaction becomes distinctly negative (down to approximately −15), reflecting the polar nature of FUR and the onset of directional electrostatic interactions, likely associated with heteroatoms in the furan ring interacting with surface functional groups. During the intermediate period (≈1–6 ns), the LJ interaction continues to strengthen, fluctuating mainly between −200 and −300, while the Coulomb interaction shows pronounced negative excursions, occasionally reaching values below −60. These strong and intermittent Coulomb minima suggest transient but significant electrostatic binding events, consistent with repeated reorientation of FUR molecules to optimize polar contacts with the VIT surface. Compared with TOL, the Coulomb contribution here is no longer negligible and plays an active role in stabilizing adsorption configurations. At longer times (>6 ns), the system exhibits large fluctuations in both interaction components. The LJ interaction remains consistently negative and occasionally reaches very deep minima (e.g., below −400 at ~18 ns), indicating periods of particularly tight packing or cooperative adsorption states. Simultaneously, the Coulomb interaction continues to oscillate over a wide range (from slightly positive values to strongly negative values approaching −80), reflecting ongoing rearrangements of polar functional groups at the interface. This sustained variability implies that, unlike the relatively static TOL–VIT adsorption, FUR maintains a more dynamic interfacial configuration.

**Figure 7:**
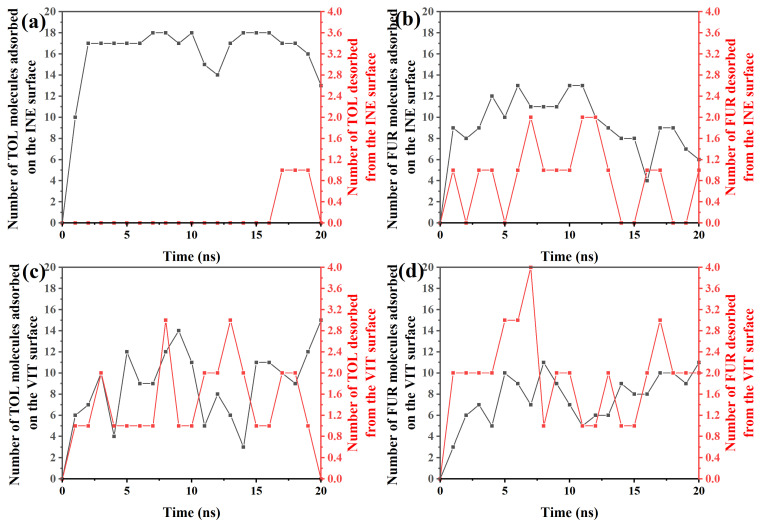
Time-dependent variation in the number of (**a**) TOL/(**b**) FUR molecules adsorbed and desorbed from the INE surface and of (**c**) TOL/(**d**) FUR molecules adsorbed and desorbed from the VIT surface.

To elucidate the dynamic adsorption behavior of organic molecules on different surfaces, the time-dependent evolution of adsorption and desorption events for TOL and FUR was systematically examined. Based on the time-resolved adsorption–desorption data, TOL exhibits a rapid and predominantly irreversible adsorption behavior on the INE surface (Figure 7a). Within the first 2 ns, the number of adsorbed TOL molecules increases sharply from 0 to 17, indicating a fast surface occupation driven by favorable interfacial interactions. From 2 to ~16 ns, the adsorption number fluctuates slightly around 17–18, while no desorption events are observed, suggesting that the adsorbed TOL molecules are kinetically trapped and form a relatively stable adsorption layer. At later stages of the simulation (17–20 ns), occasional desorption events emerge, with at most one molecule detaching from the surface, accompanied by a gradual decrease in the number of adsorbed molecules to 13 at 20 ns. This limited desorption implies that although the TOL–INE interaction is strong enough to sustain long-term adsorption, thermal fluctuations can induce partial release at extended times. Overall, the dominance of adsorption over desorption throughout the simulation highlights the strong affinity of TOL toward the INE surface and is consistent with a stable, but not completely irreversible, adsorption mechanism.

In contrast to TOL, the adsorption behavior of FUR on the INE surface is more dynamic and reversible (Figure 7b). During the initial stage (0–4 ns), the number of adsorbed FUR molecules increases modestly to 9–12, but this growth is accompanied by frequent desorption events, with 0–1 molecule detaching from the surface. This simultaneous adsorption–desorption process indicates that FUR establishes a weaker and less stable interfacial association with INE at early times, likely due to competition between surface interactions and thermal motion. From 5 to ~11 ns, the adsorbed population fluctuates between 10 and 13, while the number of desorbed molecules increases intermittently to 1–2, reflecting a quasi-dynamic equilibrium rather than persistent accumulation. At longer times (12–20 ns), a gradual decline in adsorption is observed, with the number of surface-bound FUR molecules decreasing to 6–9 and recurring desorption events continuing to occur. This trend suggests that FUR molecules do not form a stable adsorption layer on the INE surface; instead, they repeatedly exchange between the surface and the bulk phase. Overall, the pronounced fluctuations and sustained desorption highlight the relatively weak affinity of FUR for INE, consistent with a reversible adsorption mechanism dominated by transient interactions rather than long-term surface confinement.

For TOL adsorbed on the VIT surface (Figure 7c), the adsorption–desorption behavior exhibits pronounced temporal fluctuations, indicating a highly dynamic interfacial process. In the early stage (0–3 ns), the number of adsorbed TOL molecules rises rapidly from 0 to 10, accompanied by 1–2 desorption events, suggesting that TOL molecules readily approach and attach to the VIT surface but do not remain permanently bound. This rapid exchange reflects an initial competition between adsorption driving forces and thermal agitation. Between 4 and ~14 ns, the adsorption number oscillates strongly between 3 and 14, while desorption events persist at levels of 1–3 molecules. Such synchronized fluctuations imply that adsorption on the VIT surface is reversible, with frequent attachment and detachment occurring simultaneously rather than continuous accumulation. In the later stage (15–20 ns), the adsorbed population recovers and increases to 11–15, whereas desorption gradually weakens and eventually drops to zero at 20 ns. This behavior suggests that, despite the early instability, TOL molecules can ultimately establish a relatively more stable adsorption state on the VIT surface, likely driven by cumulative van der Waals interactions and improved surface packing at longer times.

For FUR on the VIT surface (Figure 7d), the adsorption–desorption process is characterized by moderate adsorption capacity but persistent molecular exchange. In the initial stage (0–3 ns), the number of adsorbed FUR molecules increases gradually from 0 to 7, while desorption events already reach 2 molecules, indicating that FUR adsorption on VIT is established early but remains weakly stabilized. This simultaneous adsorption and desorption suggests that newly adsorbed FUR molecules are prone to detach before forming a compact interfacial layer. During the intermediate period (4–14 ns), the adsorbed population fluctuates within a relatively narrow range of 5–11 molecules, accompanied by frequent desorption events (1–4 molecules). Such behavior reflects a dynamic adsorption equilibrium rather than continuous accumulation, implying limited affinity between FUR and the VIT surface. In the later stage (15–20 ns), adsorption increases slightly to 8–11 molecules, yet desorption persists at 2–3 molecules, demonstrating that even at longer times FUR molecules do not achieve a fully stable adsorption state. Overall, compared with TOL on VIT, FUR exhibits weaker adsorption stability and higher reversibility, consistent with a more labile interfacial interaction dominated by transient contacts rather than strong binding.

**Figure 8:**
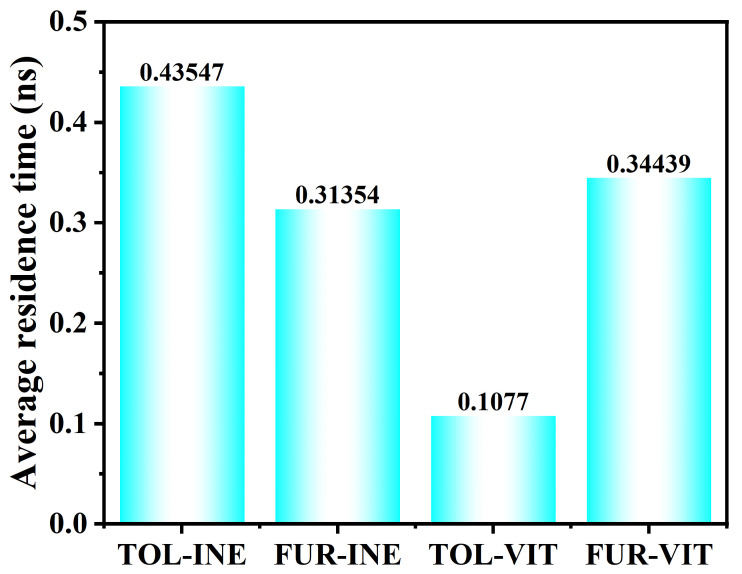
Residence time of TOL and FUR on coal surfaces in different systems.

The residence time analysis further quantifies the stability of TOL and FUR at the two mineral surfaces (Figure 8). On the INE surface, TOL exhibits a longer residence time (0.43547 ns) than FUR (0.31354 ns), indicating that TOL molecules remain attached for extended periods once adsorbed. This behavior is consistent with a more stable adsorption configuration of TOL on INE, whereas FUR shows more frequent detachment events and higher interfacial mobility. In contrast, markedly different trends are observed on the VIT surface. The residence time of TOL drops sharply to 0.1077 ns, revealing a highly transient adsorption process with rapid adsorption–desorption cycling. FUR, however, displays a substantially longer residence time on VIT (0.34439 ns), suggesting that FUR–VIT interactions are more persistent and energetically favorable than those of TOL. Overall, the residence time results highlight a clear surface selectivity: TOL preferentially stabilizes on INE, while FUR shows stronger retention on VIT, reflecting distinct molecular–surface interaction mechanisms in the two systems.

**Figure 9:**
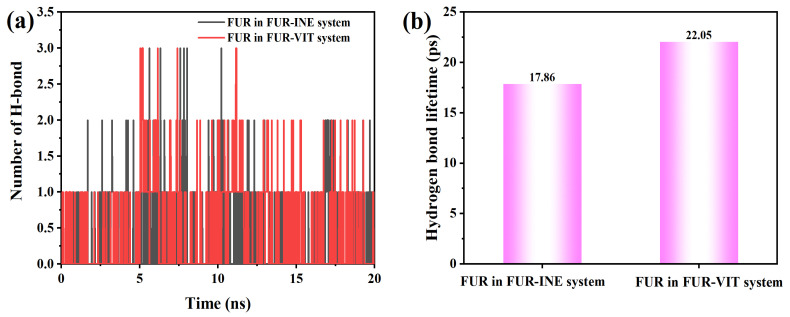
Changes in the (**a**) Number of Hydrogen Bonds Between FUR and Coal Molecules and (**b**) Hydrogen Bond Lifetime.

To further elucidate the interfacial interaction mechanism, the time evolution of hydrogen bonds formed between FUR and the INE and VIT surfaces was examined, together with their corresponding lifetimes. The hydrogen-bond statistics indicate that FUR forms only a limited number of hydrogen bonds with both INE and VIT surfaces, and these interactions appear intermittently rather than persistently over the entire simulation. For the FUR–INE system (Figure 9), the hydrogen-bond number is predominantly zero or one, with only occasional appearances of two bonds, suggesting that hydrogen bonding plays a secondary and highly transient role in stabilizing FUR at the INE interface. This interpretation is consistent with the relatively short hydrogen-bond lifetime of 17.86 ps, implying rapid formation and rupture events.

In comparison, the FUR–VIT system exhibits slightly more frequent hydrogen-bonding events, including sporadic occurrences of two to three hydrogen bonds, particularly at intermediate simulation times (Figure 9a,b). Although the instantaneous hydrogen-bond numbers remain low overall, the longer average lifetime of 22.05 ps indicates that once formed, hydrogen bonds with the VIT surface tend to persist marginally longer than those with INE. This difference suggests that hydrogen bonding contributes more effectively to the interfacial stabilization of FUR on VIT, complementing other noncovalent interactions and partially explaining the stronger interfacial affinity observed for the FUR–VIT system.

## 3. Materials and Methods

**Figure 10:**
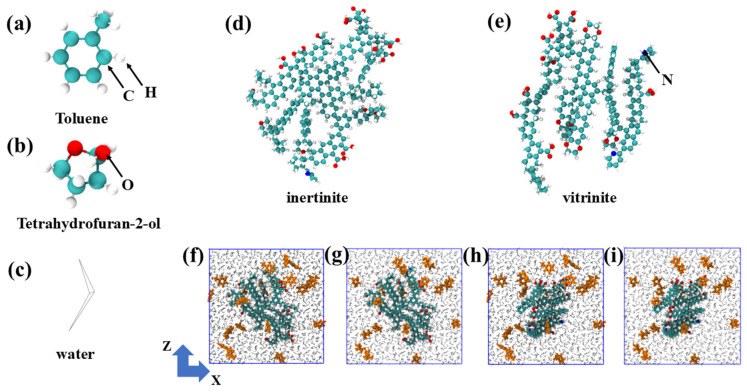
(**a**) Toluene, (**b**) Tetrahydrofuran-2-ol, and (**c**) Water molecular structures. Molecular structures of (**d**) inertinite and (**e**) vitrinite. (**f**) Initial composite system of the toluene/(**g**) tetrahydrofuran-2-ol with inertinite. (**h**) Initial composite system of the toluene/(**i**) tetrahydrofuran-2-ol with vitrinite.

The basic molecular models of toluene (TOL), tetrahydrofuran-2-ol (FUR), water molecules, inertinite (INE), and vitrinite (VIT) are illustrated in Figure 10a–e. The molecular models of vitrinite (VIT) and inertinite (INE) were constructed based on representative maceral structures. These models capture the general petrological and chemical characteristics of the two macerals, rather than specific coal samples. In particular, the vitrinite model is characterized by a higher proportion of aliphatic chains and oxygen-containing functional groups, whereas the inertinite model features a higher degree of aromatic condensation and lower oxygen content, consistent with widely accepted maceral characteristics derived from elemental analysis, FTIR, and solid-state ^13^C NMR studies. Initial maceral structures were built using an amorphous cell construction approach, in which molecular fragments reflecting the characteristic functional groups and aromaticity of each maceral were randomly packed and subsequently optimized. Energy minimization and equilibration simulations were then performed to obtain structurally stable maceral surfaces for subsequent adsorption simulations. White spheres represent H atoms, green spheres represent C atoms, red spheres represent O atoms, gray lines represent water molecules, and blue spheres represent N atoms. Organic molecules within the composite system are highlighted in orange. Inertinite and vitrinite exhibit distinct molecular characteristics. Inertinite is generally enriched in highly condensed aromatic structures with relatively low contents of oxygen-containing functional groups, resulting in a rigid and structurally stable macromolecular framework. In contrast, vitrinite contains a higher proportion of aliphatic chains and oxygen-bearing functional groups, leading to a more heterogeneous and chemically active surface. These molecular-level differences are expected to influence the strength and selectivity of organic molecule adsorption on coal surfaces. For each simulation, a single maceral model, either inertinite or vitrinite, was first constructed within the simulation domain. Twenty molecules of toluene or tetrahydrofuran-2-ol were then randomly embedded into the system, resulting in four maceral–organic composite configurations. The remaining void space was subsequently occupied by water molecules, which were added to achieve a density consistent with ambient conditions. All systems were initialized in a cubic simulation box with dimensions of 5 nm × 5 nm × 5 nm (Figure 10f–i). All simulation are performed using the GROMACS v5.0.7 software package. All the simulation details refer to the Supporting Information.

## 4. Conclusions

In this work, molecular dynamics simulations were employed to investigate the adsorption–desorption behavior of toluene (TOL) and tetrahydrofuran-2-ol (FUR) on inertinite (INE) and vitrinite (VIT) surfaces, with a focus on local interfacial interactions, molecular mobility, and dynamic stability at the coal–organic interface. It should be emphasized that the simulations describe local surface adsorption phenomena on molecular-scale maceral models, rather than quantitative adsorption in real micro- and mesoporous coal networks.

The simulations reveal a clear molecular and surface selectivity in adsorption behavior. On the INE surface, TOL exhibits the most stable adsorption, characterized by persistent surface association and a comparatively long residence time (on the order of 0.4 ns), reflecting strong non-bonded interactions between aromatic structures. In contrast, FUR on INE shows more frequent adsorption–desorption exchange and shorter surface residence, indicating weaker confinement and higher interfacial mobility.

Distinct adsorption characteristics are also observed on the VIT surface. TOL adsorption on VIT is notably less stable than on INE, with a substantially reduced residence time (approximately 0.1 ns), suggesting a highly transient surface association. By comparison, FUR displays enhanced retention on VIT, with residence times on the order of 0.3 ns, consistent with the increased contribution of electrostatic interactions and intermittent hydrogen bonding with oxygen-containing surface sites.

Hydrogen-bond analysis further indicates that hydrogen bonding plays a secondary but non-negligible role in regulating FUR interfacial dynamics. FUR forms slightly longer-lived hydrogen bonds on the VIT surface than on INE, with average lifetimes on the scale of tens of picoseconds, which contributes to the comparatively stronger surface retention on VIT despite the overall dynamic nature of adsorption.

Overall, the combined analyses of residence behavior, interaction mechanisms, and molecular mobility suggest that adsorption stability follows the general trend: TOL–INE ≫ TOL–VIT ≈ FUR–VIT > FUR–INE. These findings highlight how surface aromaticity, chemical heterogeneity, and molecular polarity jointly govern local adsorption dynamics. The molecular-scale insights obtained here provide a mechanistic basis for understanding organic matter retention and interfacial transport in coal-related energy and environmental systems and offer guidance for future multiscale studies incorporating realistic pore structures and reservoir conditions. Future studies may extend this work by incorporating explicit pore structures, mixed-maceral assemblies, and a broader range of organic species to better bridge molecular-scale insights with adsorption and transport behaviors in real coal systems. Such efforts will help improve the quantitative relevance of molecular simulations for coal-related energy and environmental applications.

## Data Availability

The original contributions presented in this study are included in the article/Supplementary Materials. Further inquiries can be directed to the corresponding author.

## Supplementary Materials

The following supporting information can be downloaded at: https://www.mdpi.com/article/10.3390/ijms27031385/s1.
