# Supplementary material for: Influence of Coal Petrology Characteristics on the Organic Matter Adsorption Properties: A Molecular Simulation Perspective
Source: Int J Mol Sci. 2026 Jan 30;27(3):1385. doi: 10.3390/ijms27031385 (PMC12898437; doi:10.3390/ijms27031385)
Supplement: Supplementary file 1 [file ijms-27-01385-s001.zip › ijms-4117946-supplementary.pdf]

Supporting information
For
Influence of Coal Petrology Characteristics on the Organic Matter Adsorption Properties: A Molecular Simulation Perspective

All molecular dynamics simulations were carried out using the GROMACS package (version 5.0.7). The interactions of organic species and coal maceral models were described by the OPLS-AA force field, while water molecules were represented using the TIP4P-2005 model, with molecular rigidity maintained through the SETTLE constraint algorithm. Non-bonded Lennard–Jones interactions between atoms were combined according to the Lorentz–Berthelot mixing rules. A cutoff distance of 1.20 nm was applied for short-range non-bonded interactions, whereas long-range electrostatic interactions were evaluated using the particle-mesh Ewald (PME) approach. All simulations employed a time step of 2 fs and periodic boundary conditions in the x, y, and z directions. Prior to dynamic simulations, the initial configurations were relaxed by energy minimization using the steepest descent method. Subsequently, each system was equilibrated in the isothermal–isobaric (NPT) ensemble for 500 ps at 323 K and 100 bar.The simulation temperature of 323 K and pressure of 100 bar were selected to approximate thermodynamic conditions relevant to deep coal formations and coalbed methane (CBM) reservoirs, rather than being purely model parameters. The applied pressure of 100 bar corresponds to a pressure-enhanced adsorption regime and is representative of subsurface coal formations, such as deep coal seams and coalbed methane reservoirs. Elevated pressure can influence adsorption–desorption dynamics by increasing molecular confinement near the surface and reducing desorption frequency. Therefore, the present simulations are designed to probe adsorption behavior under stabilized interfacial conditions. Although absolute adsorption–desorption rates may decrease at ambient pressure, the comparative adsorption behavior between different maceral surfaces and organic molecules is primarily determined by interfacial chemical

interactions, such as hydrogen bonding and π–π interactions, and is not expected to change qualitatively with pressure.During this initial equilibration stage, temperature regulation was achieved using the velocity-rescaling (V-rescale) thermostat, while pressure was maintained via the Berendsen barostat, with coupling time constants set to 0.1 ps and 2 ps, respectively. After this pre-equilibration step, extended NPT production runs with durations of no less than 200 ns were performed under identical thermodynamic conditions. In the production phase, temperature and pressure were controlled using the Nosé–Hoover thermostat and the Parrinello–Rahman barostat, respectively, with corresponding relaxation times of 2 ps for temperature and 4 ps for pressure. An isotropic scheme was applied for pressure coupling throughout the simulations.

In this study, adsorption and desorption events were defined based on the distance between organic molecules and the maceral surface. An organic molecule was considered adsorbed when any heavy atom of the molecule was located within 5 Å of the maceral surface. Conversely, a molecule was regarded as desorbed once its distance exceeded 5 Å. This distance-based criterion has been widely adopted in molecular dynamics studies of interfacial adsorption and provides a consistent measure for analyzing adsorption–desorption dynamics. It should be noted that the maceral models employed in this study are idealised molecular representations without explicit pore networks. This modelling strategy is adopted to isolate and examine the intrinsic interfacial interactions between organic molecules and maceral surfaces at the molecular scale. While pore texture and porosity play important roles in bulk adsorption behavior, their effects are deliberately excluded here in order to avoid geometric and transport-related complexities and to focus on chemical interaction mechanisms governing adsorption selectivity.
